# POCUS vs. CT in acute cholecystitis: are we really reducing ED length of stay?

**DOI:** 10.1186/s13049-025-01367-6

**Published:** 2025-03-26

**Authors:** Caglar Kuas, Volkan Ercan, Mustafa Emin Canakci

**Affiliations:** Eskisehir Osmangazi University, Eskişehir, Turkey

**Dear Editor**,

We have read with great interest the recent article, *“Impact of a POCUS-first versus CT-first Open Access approach on emergency department length of stay and time to surgical consultation in patients with acute cholecystitis: a retrospective study”* by Chien-Tai Huang et al. [1]. We believe that this research makes a valuable contribution to the discussion on the impact of point-of-care ultrasound (POCUS) and computed tomography (CT) in the management of acute cholecystitis. However, certain methodological aspects warrant further clarification.

One of our primary concerns is that the study exclusively evaluates patients with Grade I (mild) acute cholecystitis based on the Tokyo Guidelines. However, a significant proportion of patients presenting to the emergency department with cholecystitis are classified as Grade II (moderate) or Grade III (severe), and the diagnostic and management processes for these patients are considerably more complex [2, 3]. In particular, Grade II-III cases are characterised by more pronounced inflammation and an elevated risk of complications [4], which may have implications for the diagnostic accuracy and clinical utility of imaging modalities. The effectiveness of POCUS in this patient population must be assessed to draw definitive conclusions regarding its overall clinical utility. The study’s inclusion of only Grade I patients limits the generalisability of its findings, and further clarification is needed on why a broader patient population was excluded.

Furthermore, the study states that all patients underwent CT imaging. However, in clinical practice, ultrasonography (US) is generally the first-line imaging modality, while CT is typically reserved for cases where additional differential diagnoses are suspected or for assessing complications [5, 6]. Routinely performing CT on all patients may lead to unnecessary radiation exposure and prolonged ED stays. The study does not provide a clear justification for why CT was used as a mandatory diagnostic tool instead of ultrasonography.

It is also worthy of note that the emergency department length of stay (ED-LOS) was reported to be considerably protracted, with no significant disparities observed between the various imaging modalities. While point-of-care ultrasound (POCUS) is theoretically expected to accelerate patient management by enabling early diagnosis, the results demonstrate that this is not reflected in clinical practice. In the patient group where POCUS was performed within 60 min, the median time to surgical consultation was reported as 5.7 h, and the ED-LOS was 26.9 h. This finding indicates that factors external to imaging, such as the availability of the surgical team, delays in admission processes, or inefficiencies in surgical scheduling, play a significant role in patient flow management. Should the overall ED-LOS remain unchanged despite the acceleration of the diagnostic process, further analysis of in-hospital organisational factors is warranted. Furthermore, the study defines a 60-minute threshold for POCUS, whereas the corresponding threshold for CT is set at 120 min. However, the rationale behind these thresholds and how they were determined is not explicitly stated.

We believe this study provides valuable insights into the literature. Future prospective studies assessing the impact of POCUS and CT on time management, particularly in Grade II and III patients, would be beneficial.

## Data Availability

No datasets were generated or analysed during the current study.
